# Effect of support supervision on maternal and newborn health services and practices in Rural Eastern Uganda

**DOI:** 10.1080/16549716.2017.1345496

**Published:** 2017-08-18

**Authors:** Angela N. Kisakye, Rornald Muhumuza Kananura, Elizabeth Ekirapa-Kiracho, John Bua, Martha Akulume, Gertrude Namazzi, Suzanne Namusoke Kiwanuka

**Affiliations:** ^a^ Department of Health Policy, Planning and Management, Makerere University School of Public Health, Kampala, Uganda

**Keywords:** Support supervision, implementation science, human resource, health workers, maternal and newborn services

## Abstract

**Background**: Support supervision is one of the strategies used to check the quality of services provided at health facilities. From 2013 to 2015, Makerere University School of Public Health strengthened support supervision in the district of Kibuku, Kamuli and Pallisa in Eastern Uganda to improve the quality of maternal and newborn services.

**Objective**: This article assesses quality improvements in maternal and newborn care services and practices during this period.

**Methods**: District management teams were trained for two days on how to conduct the supportive supervision. Teams were then allocated particular facilities, which they consistently visited every quarter. During each visit, teams scored the performance of each facility based on checklists; feedback and corrective actions were implemented. Support supervision focused on maternal health services, newborn care services, human resources, laboratory services, availability of Information, education and communication materials and infrastructure. Support supervision reports and checklists from a total of 28 health facilities, each with at least three support supervision visits, were analyzed for this study and 20 key-informant interviews conducted.

**Results**: There was noticeable improvement in maternal and newborn services. For instance, across the first, second and third quarters, availability of parenteral oxytocin increased from 57% to 75% and then to 82%. Removal of retained products increased from 14% to 50% to 54%, respectively. There was perceived improvement in the use of standards and guidelines for emergency obstetric care and quality of care provided. Qualitatively, three themes were identified that promote the success of supportive supervision: changes in the support supervision style, changes in the adherence to clinical standards and guidelines, and multi-stakeholder engagement.

**Conclusion**: Support supervision helped district health managers to identify and address maternal and newborn service-delivery gaps. However, issues beyond the jurisdiction of district health managers and facility managers may require additional interventions beyond supportive supervision.

## Background

Life-threatening complications during pregnancy, delivery and the postpartum period are commonly encountered disproportionately in low- and middle-income countries [1,2]. The health complications of pregnant women, mothers and newborns cannot be mitigated without due attention to the quality of care [3,4]. Skilled delivery has long been indicated as the most effective intervention; but with the prevalent shortage of life-saving products and equipment, health workers cannot be expected to literally save lives with their bare hands [5]. The availability of Emergency Obstetric Care (EmOC) services is a major strategy to ensure that pregnant women and newborns have access to well-functioning facilities that include a wide range of maternal and newborn services [1,2,6,7]. The basic EmOC services that should always be at the facilities include administration of parenteral antibiotics, perinatal antibiotics, oxytocic drugs and anticonvulsants, manual removal of placenta, removal of retained products and assisted vaginal delivery [1]. Facilities that provide comprehensive services should perform all basic EmOC services, as well as cesarean section and provision of blood transfusion [2,7].

In Uganda, the health care system has long been characterized by poor health worker distribution, inadequate skills sets and skill mix, insufficient drugs and basic supplies, poor infection-control practices and overall weakness in the management of service points [8,9]. These have contributed to the persisting high levels of maternal and newborn deaths, estimated at 438 per 100,000 live births and 27 per 1000 live births, respectively. The availability of EmOC services, availability of skilled health workers and their knowledge in the provision of maternal and newborn care services are therefore essential components for improved maternal and newborn care services. In addition, the EmOC package should include infection prevention and management, monitoring and management of labor using partographs, active management of third stage of labor, infant thermal protection, feeding and HIV prevention [1].

Countries with low maternal mortality rate (MMR) have a high proportion of births attended by skilled attendants and almost universal access to high-quality EmOC in case of obstetric emergencies [2]. However, most facilitates in Uganda, like many Sub-Saharan African countries, are not always able to provide emergency obstetric care. This compromises the quality of care mothers and their newborns receive during pregnancy, at the time of delivery and postpartum. Supportive supervision is an avenue used to monitor the quality of healthcare delivered by health workers who have limited training. In this way, it enhances the quality of services provided at the health facilities [9]. However, this support is not always provided in many developing countries [9]. Moreover, those that conduct support supervisions frequently lack technical and managerial skills and may have no authority to resolve service delivery problems [9].

Available evidence highlights the importance of support supervision in strengthening relationships within the system, identification and resolution of problems, optimizing the allocation of resources, motivating health workers, promoting the use of standard procedures, and teamwork [10,11]. Support supervision also provides opportunities for regular feedback and therefore promotes skill building through appropriate strategies, such as training and mentorships [12,13]. Inadequate support supervision has been cited as a key contributor to health worker shortages in low- and middle-income countries. It is usually conducted with the assistance of senior colleagues who have amassed experience over the years [14]. The conduction of supportive supervision is threatened by work overload and ethos among clinicians who think that the supervision portrays them as incompetent [15]. Supportive supervision approaches have been reported to make practitioners feel sustained and revitalized because they are not ‘fault-finding’ missions [16].

To improve the quality of services at health facilities, Makerere University School of Public Health implemented the Maternal and Neonatal Implementation for Equitable Systems (MANIFEST) project. The MANIFEST project provided team-based support supervision to facilities in the district of Kibuku, Kamuli and Pallisa in Eastern Uganda for a period of three years (January 2013–December 2015) to help strengthen support supervision and improve maternal and newborn health outcomes.

### Description of MANIFEST support supervision

In each district, three supportive supervision teams were constituted. These comprised researchers from Makerere University School of Public Health, district health teams and specialists (obstetricians and pediatricians) from the Ministry of Health and regional hospitals. The district health team was composed of district health officers (DHOs), maternal child health focal persons, senior midwives and senior nurses from select facilities. Supportive supervision teams conducted quarterly visits. During each visit, the teams initially reviewed the gaps identified at previous visits with the facility providers. At the facilities, a checklist of indicators was used to assess the availability of maternal and newborn services, and services available at different facility levels. The checklist used at Health Center II and Health Center III level consisted of indicators that focused on human resource numbers and absenteeism, provision of emergency obstetric and newborn care, availability of essential laboratory services, availability of client records and information education communication (IEC) materials, availability of adequate physical infrastructure, availability of infection control guidelines, facility referral readiness, availability and use of partographs, as well as follow up of the agreed action points of the previous support supervision. In addition to all the above indicators, provision of cesarean section and blood transfusion were included for the Health Center IV and hospital checklists. The checklists provided scores for facility performance across each aspect assessed. Each facility’s performance was based on the proportion of marks obtained across each category out of the maximum possible scores obtained at each visit/total possible scores on the checklist.

The team provided feedback on performance, gave technical updates or guidelines and carried out on-the-job learning drills to improve skills. Supervisors and health workers jointly identified opportunities for improvement through observation and review of health facility needs. In addition, joint problem solving and follow up of action points were undertaken in a participatory manner with health workers and the district health office, who were actively involved in resolving problem areas. At the end of each facility visit, the checklist, with its identified gaps, plans and proposed action plans, was submitted at the district level. The district health manager then synthesized these checklists and produced a support supervision report, which was shared with the research team through quarterly review meetings.

This article highlights improvements in maternal and newborn care services and practices as a result of support supervision under MANIFEST.

## Methods

### Study design

A mixed-≈methods study was conducted in three districts of Eastern Uganda under the MANIFEST project. A total of 28 health facilities, which received a minimum of three support supervision visits, were considered for this study. All 28 facilities were government health centers: two level II health centers, 22 level III health centers, two level IV health centers and one hospital. Details on the study design are described in the study protocol [17] of this supplement.

### Data collection and analysis

#### Quantitative data

Support supervision reports submitted by the districts were reviewed and data for facilities that had at least three consecutive support supervisions visits were included. For each facility, data on performance across the indicators were entered in an Excel spreadsheet and then exported to STATA for analysis. A t-test was used to estimate the mean difference in facility performance between the first support supervision and the two subsequent scores (quarters two and three). In addition, the data from the health facility checklists was analyzed for changes in the availability of an adequate number of trained staff, equipment, drugs and supplies, IEC materials and infrastructure via the use of proportions/percentages.

#### Qualitative data

Twenty key informant (KI) interviews were conducted with seven health workers, seven health facility in-charges, three DHOs and three maternal and newborn focal persons. These were purposively selected because they were key people during the supportive supervision. The KI interviews were recorded using audio recorders with consent from the participants. The qualitative data was transcribed and reconciled with notes recorded during the interviews and then analyzed using thematic analysis following the six steps recommended by Braun and Clarke [18]. The data from the transcripts was analyzed manually by reading and rereading of the data to identify themes.

## Results

### Changes in facility performance

Overall, facility performance improved with the MANIFEST support supervision intervention. Most facilities scored highly at the third support supervision (mean = 61.3 and range from 28 to 93) compared to the first-quarter scores (mean = 43.9 and range from 8 to 79). The difference between the first- and third-quarter scores was 17.4 and was statistically significant (t = 3.16, p = 0.0015). However, about 10% (4) facilities showed a decline in performance. Table 1 summarizes the facility performance scores across the first and the third supervision scores.

**Table 1.** Comparison between baseline and endline supervision scores.

Variable	Mean score (%)	Minimum score (%)	Maximum score (%)
Baseline score	43.92	8	79
2^nd^ score	49.92	5	82
3^rd^ score	61.28	28	93
**Difference between baseline score and 3^rd^ supervisory score**	6.00 (t = 3.16, p = 0.0015)		
**Difference between baseline score and 3rd supervisory score**	17.36 (t = 8.50, p = 0.001)		

Notes: Total number of health facilities is 28.

### Changes in the availability of maternal health services

Results from the first- and third-quarter supervision visits indicated an increase in delivery and post-abortion care services. For instance, across the first, second and third quarters, the availability of parenteral oxytocin increased from 57% to 75% to 82%, respectively; assisted vaginal deliveries increased from 7% to 11% to 21%, respectively; manual removal of retained products increased from 14% to 50% to 54%, respectively; and the provision of vitamin K increased from 29% to 21% to 43%, respectively.

## Changes in newborn care services and practices

**Figure 1.**
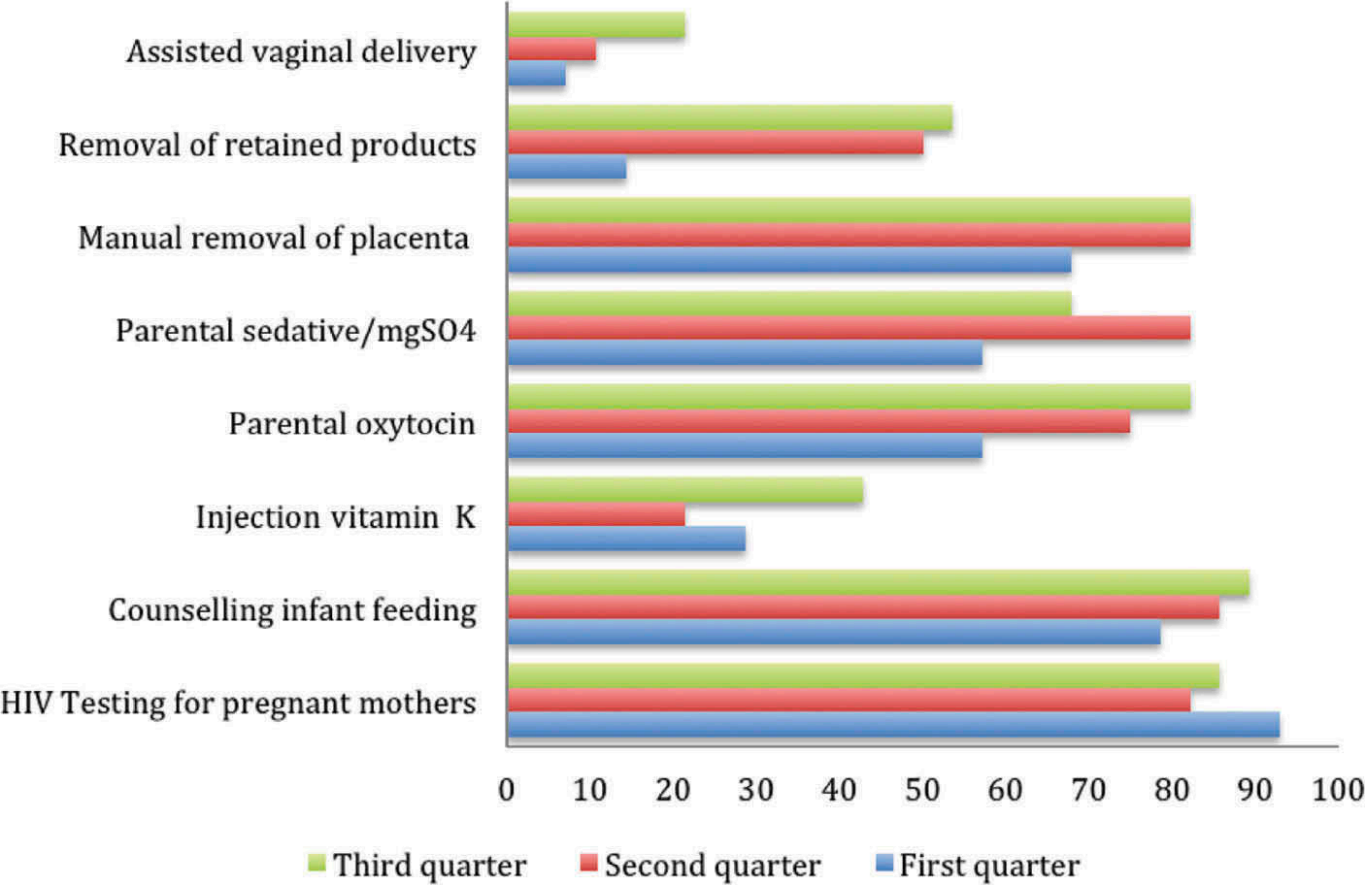
Changes in newborn care services and practices.

During the implementation period, there was an improvement in the provision of essential newborn care services including feeding the baby within one hour, examination of newborns, cord care promotion and keeping the baby warm. Promotion of cord care and feeding the baby within an hour increased from 82.1% in the first quarter to 89.3% and 96.4% in the second and third quarter, respectively. However, there was no improvement in the provision of antiretroviral drugs to mothers and babies in need, or in the provision of tetracycline eye ointment to newborns. Figure 1 summarizes changes in the availability of newborn care services.

### Changes in the availability of laboratory services

**Figure 2.**
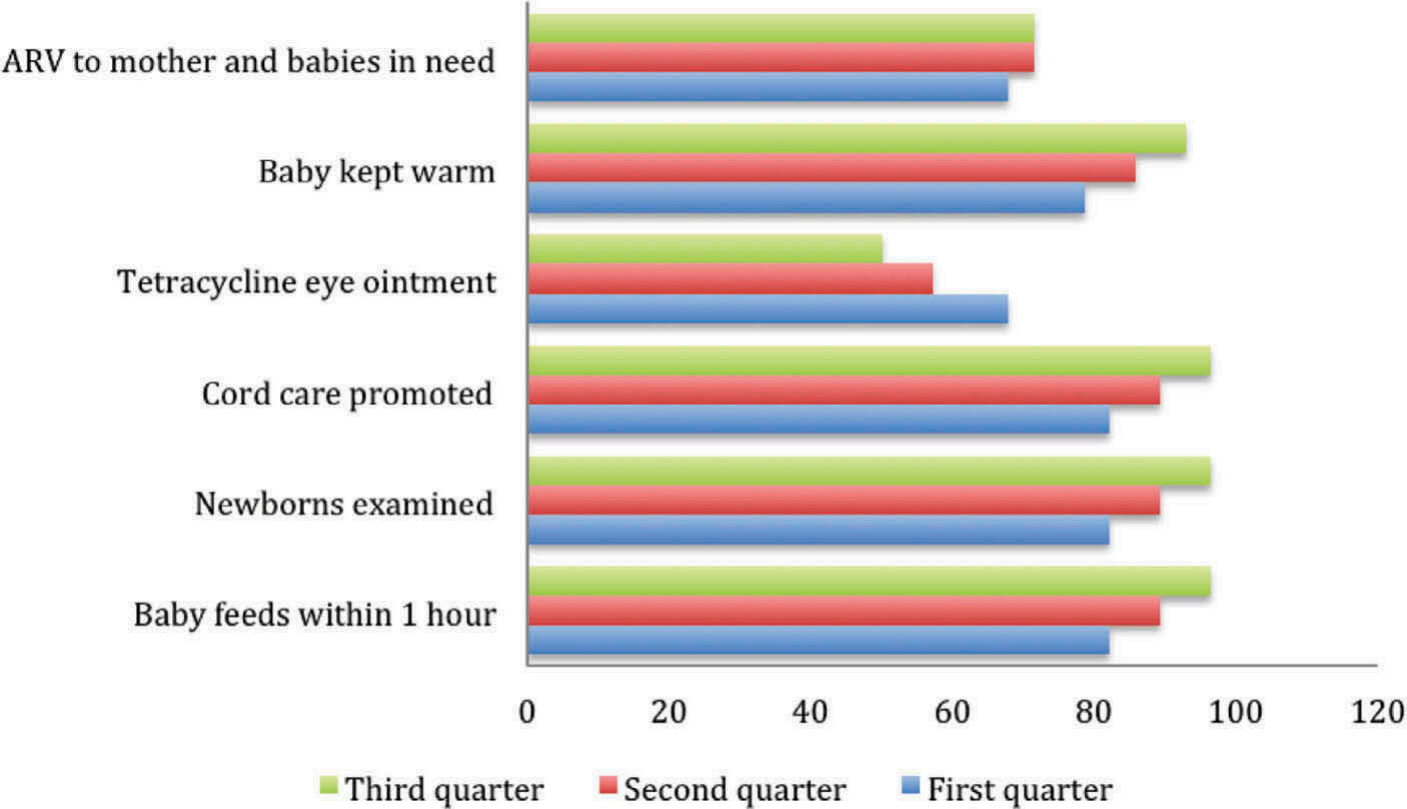
Changes in laboratory services.

Data across the three support supervision points revealed that there was generally no improvement in the laboratory services. Comparing the first and third quarter, facilities that had urine testing for pregnant women reduced from 17.9% to 7.1%, while facilities that conducted blood grouping and cross-matching reduced from 14.3% to 10.7%. However, facilities with syphilis testing increased from 32.1% to 71.4% across the three quarters. Figure 2 summarizes changes in the availability of laboratory services.

### Changes in the availability of records and IEC materials

There was high variability in the availability of records and IEC materials at almost all health facilities throughout the supportive supervision period. For instance, across the first, second and third quarters, Antenatal care (ANC) registers were increased from 92.9% to 964% to 96.4%. Maternity registers were available in 85.7%, 89.3% and 85.7% of the facilities in the first, second and third quarter, respectively. However, discharge forms, postnatal care (PNC) forms, family planning forms and ANC cards were constantly below 20% across all facilities in the first, second and third quarters, while referral forms were continually below 35% throughout the three quarters. In addition, availability of management protocols and guidelines at health facilities declined from 57.1% to 46.4% between the first and third quarter.

### Changes in the availability of infrastructure

**Figure 3.**
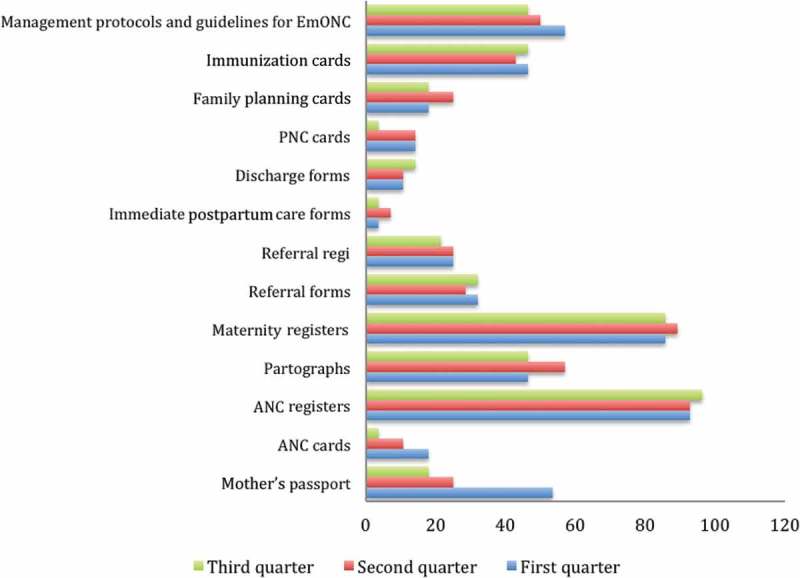
Changes in availability of maternal and health services. BS: blood sample; MPs: malaria parasite.

Facilities with a basic laboratory increased from 67.9% in the first quarter to 75% in the second and third quarters (Figure 3); delivery beds increased from 60.7% in the first quarter to 71.4% in the second and third quarters, while ANC and PNC for pregnant women and mothers increased from 82.7% in the first quarter to 92.9% in the second and third quarter, respectively. Across the first, second and third quarters, basic pharmacy services increased from 60.7% to 64.3% to 78.6%; lighting in the maternity ward increased from 60.7% to 64.3% to 67.9%. Regarding infection control, only 43% of the health facilities had running water during the third support supervision; a reduction of 10% compared to the first-quarter support supervision. The number of facilities with placenta pits and waste segregation bins increased from 78.6% in the first quarter to 89.3% in the second and third quarter, respectively. Referral readiness was lacking throughout the three quarters. Availability of 24-hour ambulatory services and human resources to manage ambulances was below 20% across all facilities.

### Changes in health worker availability

The medical officers working at the hospital and two Health Center IVs were not available on site during the three support supervision quarters. The presence of midwives for their work shifts increased from 43.8% to 56.3% between the first and third quarter, respectively. However, the availability of nursing officers reduced from 50% to 25% in the second and third quarters. At least half of the enrolled nurses were present at their work stations throughout the implementation period. 

### Changes in the support supervision style

**Figure 4.**
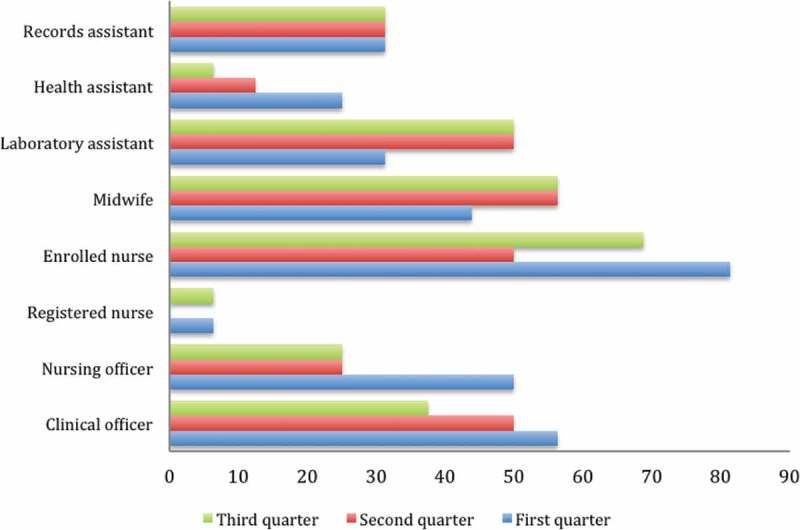
Changes in availability of IEC and record materials.

Analysis of qualitative data revealed three major themes: changes in the support supervision style; changes in the adherence to clinical standards and guidelines; and multi-stakeholder engagement (Figure 4).

### Changes in supervision style

Key informants noted that the support supervision under MANIFEST differed greatly from what they had done in the past. They acknowledged that previous support supervision was cursory, and mostly blamed health workers for identified weaknesses. Moreover, they appreciated the support supervision tool used by MANIFEST because it enabled systematic assessment of facilities and follow up on action plans.

‘We used not to go to those health facilities and spend hours there. We would just go look around; it was almost fault finding. But when MANIFEST came we developed a tool together with them – but those days before MANIFEST, we could just go rushing. Actually we didn’t have a tool for supervision. But these tools were developed to guide us to identify those key areas we felt we needed to improve and we needed to keep on tracking.’ (Focal person, Kamuli District)

‘There is a change, because … formerly when these people would come to supervise us, it’s like they were policemen and you know we could even fear [them]. When you hear people are coming for supervision, you would rather disappear before they get you. But now everything has changed; they come and it’s a friendly supervision. Somebody comes to support you, not to blame you for the mistake…’ (Facility in-charge, Pallisa District)

**Figure 5.**
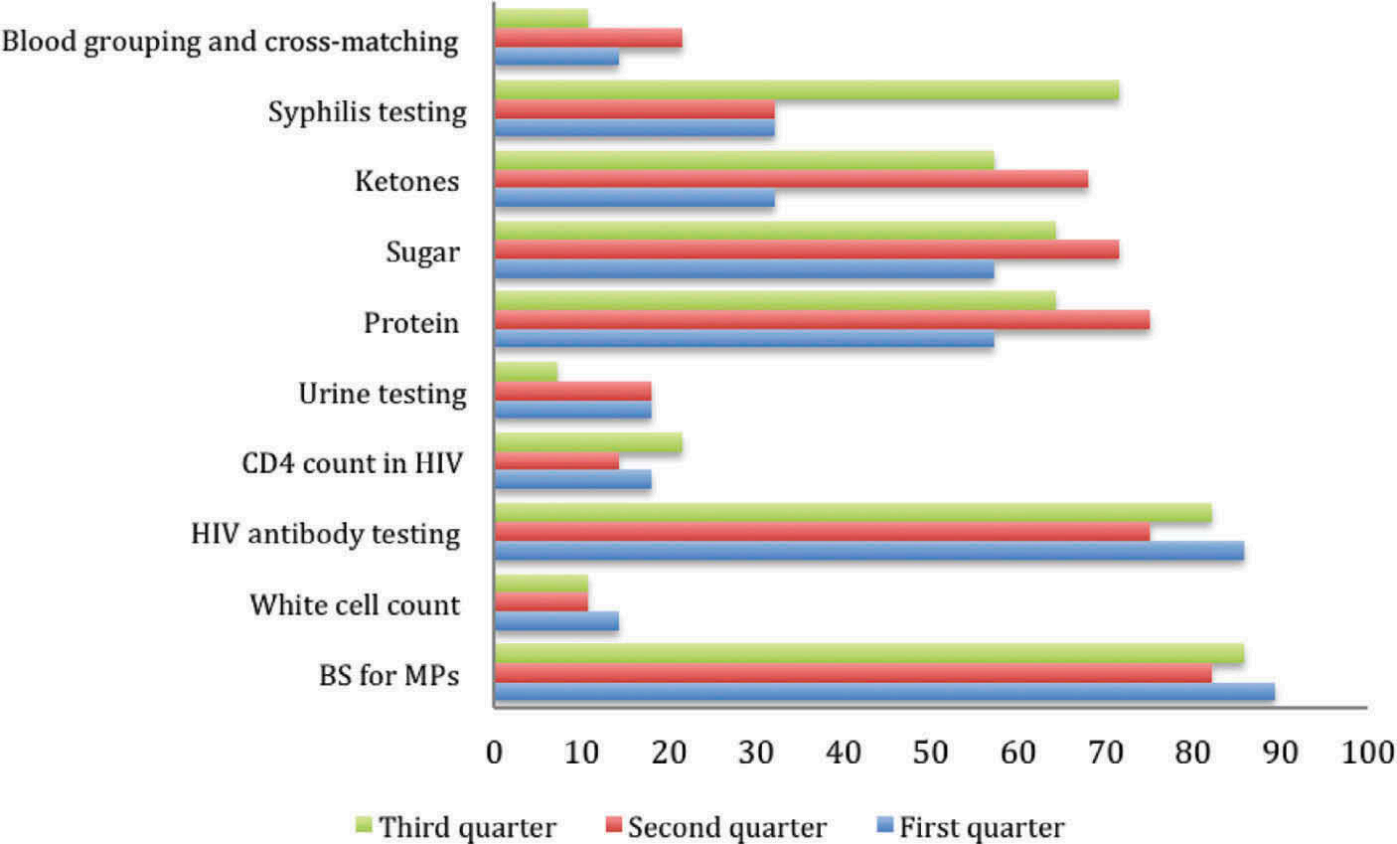
Changes in availability of infrastructure.

**Figure 6.**
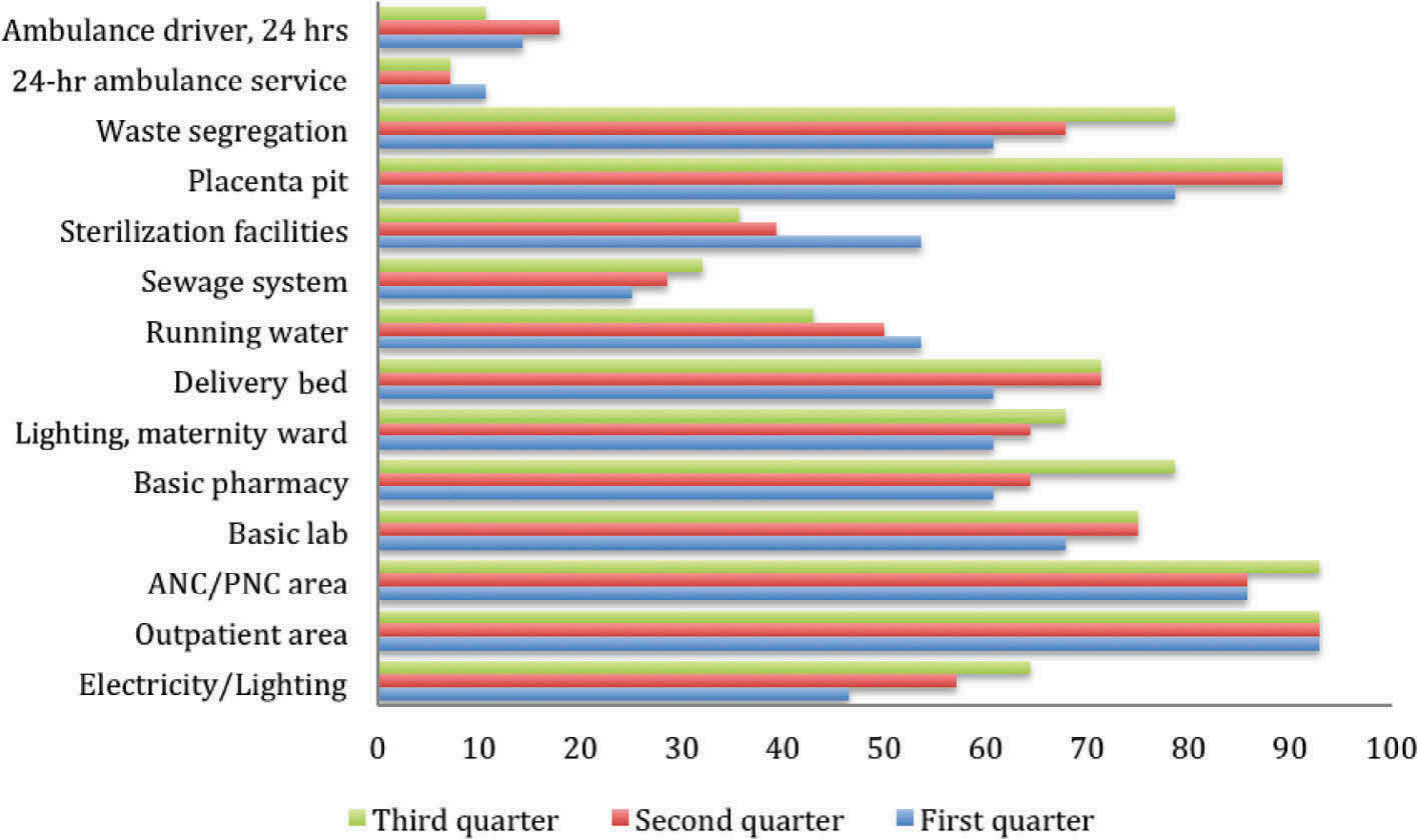
Changes in staffing availability.

They noted that this new approach to support supervision was premised on bridging gaps identified at facilities and empowering the concerned people to address them. The feedback from each support supervision visit was used to ensure that proper actions were taken at the facilities and beyond. The quantitative results indicated that in the beginning, health facility gaps included lack of essential drugs and equipment for maternal health, poor infection control, and poor infrastructure (see Figures 1, 5 and 6). Because the support supervision was conducted together with the district health leaders, the appropriate actions and responsibilities were assigned to the responsible parties. For instance, the DHO’s office was able to appreciate each facility’s gaps, thus making it easy for them to address the gaps or to lobby the health development partners. This also strengthened communication between the district health workers and health facilities, thus building teamwork.

‘A facility like ours here didn’t even have light, but because of the support supervision where the district leaders came and witnessed the problem we have, we managed to get solar. Before, we used to use torches – even your phone. A mother has a tear, of course the torch is not bright, you’re not seeing, the mother keeps bleeding, you go expel, yet there is a tear somewhere and the phone cannot operate…’ (In-charge, Pallisa District)

‘We identify their weakness and the next day in the debriefing (at district level), whatever we have to address at a DHO’s office has to be addressed. For example, yesterday there was a problem [with] a fridge in one of the health centers and it was identified as a major problem, so the next day we moved the fridge from the H/C II to the H/C III. So you see the support supervision now adds value.’ (District team member, Kibuku District)

‘…but ever since [the] MANIFEST project started, we have been having regular support supervision and most of the problems identified during support supervision have been addressed.’ (Facility in-charge, Kibuku District)

### Changes in adherence to clinical standards and guidelines

The first support supervision revealed inadequate use of standards and guidelines for EmOC, such as partographs. Such information enabled the delivery of a focused training program on the use of partographs, identification of danger signs and management of high-risk babies. The health workers expressed appreciation for the value of filling partographs and, consequently, their use for monitoring labour improved. This change was specifically due to the feedback given on performance at each support supervision visit, as indicated below.

‘It’s like we were not monitoring mothers on partographs. But when they came in, they [supervisors] could let us know that every mother, at least, even if she is coming in the second stage of labor, is supposed to be monitored on partographs. So we started using partography on every mother who comes and up to now, at least, we are using them.’ (Nursing officer, Kamuli District)

‘Previously there was a habit of filling the partograph after delivering a mother. Some would just sit and fill because it is a requirement, you know. Nowadays they know that partographs are filled as they monitor the mother. Now we have 100%. Previously, I think we were at 30% or 20%.’ (In-charge, Kamuli District)

### Multi-stakeholder engagement

The changes in some of the facility services are attributed to the involvement of a variety of stakeholders, who included DHOs, health subdistrict in-charges and the facility in-charges, which was different from the previous support supervision activities. This approach was perceived to have enhanced the relationship between the health workers and the supervisors, as opposed to the previous support supervisions. The health workers indicated that, previously, they preferred to call in absent before supervisory visits because their supervisors blamed them for the gaps at the facilities, instead of supporting them to address the gaps:

‘…We didn’t have a tool for supervision, but these tools were developed to guide us to identify those key areas we felt we needed to improve and we needed to keep on tracking.’ (Focal person, Kamuli District)

## Discussion

This project highlights the importance of support supervision for strengthening the health system in rural communities. The support supervision approach enabled wide stakeholder involvement, inclusive problem solving and identification of gaps/weaknesses at the facilities, which ensured that appropriate actions were taken. The approach also enabled sharing of facility service delivery gaps with the district leaders and encouraged openness by the health workers about challenges in the facility. Armed with information on facility gaps, the district health leadership was able to lobby health development partners to provide necessary support. A systematic review of support supervision revealed the importance of support supervision in increasing job satisfaction and health workers’ motivation [9]. Prior to MANIFEST, the support supervision visits were rare, and, when they occurred, were in the form of negative feedback and blaming of staff. The MANIFEST approach demonstrated that it is possible to have positive and collegial relationships between health workers and supervisors. The changes in the use of support supervision in improving the quality of maternal and newborn care services has also been reported by other studies conducted in low-income countries [10,12–14].

As a result of supportive supervision, there were some changes in the quality of the maternal health care services provided. Health workers were able to manage maternal and newborn complications. The use of facility standards, such as use of partographs in monitoring labor, improved. This was because during support supervision, health workers were observed and taught how to monitor the progress of labor. Following clinical guides is critical for health facilities to provide quality maternity and newborn care services.

Furthermore, there was an improvement in the availability of delivery services, such as parenteral antibiotics, parenteral oxytocin and parenteral sedative/mgSO4. PNC services, such as manual removal of placenta, removal of retained product, and vacuum extraction, also increased. Unfortunately, magnesium sulphate, which is critical for the prevention and treatment of preeclampsia; oxytocin, which is important for the prevention of postpartum hemorrhage (PPH) [3,19]; as well as Vitamin K were only minimally available in the health facilities. This could be attributed to inadequate supply. Regrettably, the inability to provide critical drugs to women during delivery can lead to dire consequences for both the mother and the unborn baby. For instance, seizures in women with preeclampsia or eclampsia cannot be prevented without administration of magnesium sulphate. In addition, fetal neuroprotection before anticipated early preterm (less than 32 weeks of gestation) delivery and short-term prolongation of pregnancy (up to 48 hours) to allow for the administration of antenatal corticosteroids in pregnant women who are at risk of preterm delivery within seven days cannot be implemented if an essential drug such as magnesium sulphate is unavailable [20]. Studies have indicated that the main factors identified for poor quality of care are the failure to offer 24-hour services, lack of drugs and supplies, and low competence of birth attendants [3]. It is encouraging to note that support supervision improved some of these factors, such as the availability of critical drugs and the ability of health workers to provide emergency services.

The lack of robust preventive diagnostic services, such as blood grouping, cross-matching and urine analysis, highlights a persistent critical gap in maternal services and limits the management of mothers at risk. These services should be available at all levels where mothers give birth, since hemorrhage and preeclampsia are major causes of maternal deaths. Available evidence indicates that at least 60% of maternal and newborn deaths occur in the postpartum period [4,7], usually the first day after birth, and their management falls within the skilled attendance or emergency care strategies [7].

Availability of health workers is one of the most critical elements for provision of quality maternal and newborn care services. Although there was an improvement in the availability of midwives at the health facilities during the support supervision visits, doctors at the Health Center IVs and hospitals opted not to attend any of the support supervision sessions, perceiving themselves to be above being supervised alongside lower cadre. The hierarchical gap between medical doctors and lower cadres in these settings threatens good teamwork and ultimately the quality of services. The most available cadre of health workers were enrolled nurses, yet the government wants to phase them out.

Despite the support supervision, some professional skills remained poor. For instance, the ability to conduct assisted vaginal delivery and removal of retained products remained low throughout the implementation period. The provision of essential newborn care services improved noticeably. However, there is still a gap in the provision of these services, since they were not provided in all health facilities, despite the fact that they are essential for improved newborn health and survival [14].

There were improvements in the availability of infrastructure, such as lighting and delivery beds. However, there were minimal improvements in the availability of other infrastructure, such as laboratory, basic pharmacy, maternity and delivery wards. Infrastructure improvement was poor because health workers had little control over infrastructure development.

Regarding infection control, placenta pits were constructed in facilities that did not previously have them, and waste segregation improved. This shows that improvements can be achieved at service delivery level with minimal inputs for maternal and newborn care. However, less than a half of the facilities had running water. The lack of running water presents a great challenge to health workers who need to conduct their services in an aseptic environment. This issue is better addressed by district authorities, but often bureaucracy hinders quick solutions within districts and cripples service delivery.

Major and persistent gaps were identified in referral readiness. For instance, one hospital and only one Health Center IV had a running ambulance. Issues of referral readiness may not be within the power of districts to address, and may require intervention at a higher level, such as by the Ministry of Health.

### Strengths and limitations

The strength of this study is that it assessed changes in services based on a checklist developed in collaboration with the district health workers and managers to assess services intended to be delivered at each level. The 15 health facilities were excluded because they did not have support supervision records beyond three subsequent visits. However, the overrepresentation of Health Center III (HCIII) further strengthens the findings, because not only are most maternal and newborn services delivered at this level, but systems weaknesses are also magnified. Since the study was able to track changes across quarters, it also enabled decision makers to acknowledge that decisions such as procurement of equipment, drugs and availing space were beyond the control of the health workers, yet shortcomings in these aspects hamper improvement in certain areas. The project was not without limitations. For instance, the health workers could have changed behavior because they were being observed. Poor record keeping at many of the health facilities was also a major limitation, as well as the lack of a comparison group.

## Conclusion

This project reveals that support supervision helps district health managers to identify and address maternal and newborn service delivery gaps. Overall, district leaders and facility managers are able to effect changes within their sphere of influence. However, some other issues, such as availability of drugs, supplies, equipment and infrastructure, which are beyond the jurisdiction of district health managers and facility managers, may not be addressed effectively. Therefore, there are still a number of supply-side challenges that higher-level decision makers must address in order to improve services. These include staffing, availability of drugs, laboratory-testing kits and other equipment for identification and management of complications. Lessons from the MANIFEST project should be used by other projects or studies to take supportive supervision to another level, which focuses on the gains and strengths of supportive supervision and how to sustain these in the long run.

## Paper context

From 2013 to 2015, the MANIFEST project strengthened support supervision in the districts of Kibuku, Kamuli and Pallisa in Eastern Uganda to improve the quality of maternal and newborn services. This article shares the changes in maternal and newborn care services during the support supervision period.
